# Predictors of postoperative recurrence in locally advanced gastric cancer patients achieving pathological complete response after neoadjuvant therapy: a matched case-control study

**DOI:** 10.3389/fonc.2025.1717605

**Published:** 2026-01-12

**Authors:** Zhiqiang Jiang, Jiankai Lyu, Xiliang Cong, Na Xu, Ziyan Bai, Zhouyi Zhao, Siyun Fu, Baozhen Ma, Lingdi Zhao

**Affiliations:** 1 Department of General Surgery, the Affiliated Cancer Hospital of Zhengzhou University & Henan Cancer Hospital, Zhengzhou, China; 2 Endoscopy Center, the Affiliated Cancer Hospital of Zhengzhou University & Henan Cancer Hospital, Zhengzhou, China; 3 Department of Immunotherapy, the Affiliated Cancer Hospital of Zhengzhou University & Henan Cancer Hospital, Zhengzhou, China

**Keywords:** complete pathological response, gastric or gastroesophageal junction cancer, neoadjuvant, prognostic factors, recurrence

## Abstract

**Background:**

Achieving a pathological complete response (pCR) after neoadjuvant therapy is a favorable prognostic factor in locally advanced gastric/gastroesophageal junction cancer. However, recurrence still occurs in a subset of these patients. This study aimed to identify clinical factors associated with recurrence in patients who attained pCR.

**Methods:**

This retrospective study included 16 patients with recurrence early after achieving pCR. Each case was matched with two controls (non-recurrence) based on age and sex. Clinical characteristics were compared, and factors with *P* ≤ 0.1 in univariate analysis were included in a subsequent multivariate logistic regression analysis to identify independent predictors.

**Results:**

Univariate analysis identified significant differences between recurrence and non-recurrence groups in tumor grade, neoadjuvant therapy regimen, PD-L1 expression, and preoperative CEA levels. Multivariate analysis established clinical T stage and neoadjuvant regimen as independent prognostic factors for recurrence. Specifically, compared to patients with cT4 stage and those receiving neoadjuvant chemotherapy, patients with cT3 stage (P=0.035) and those receiving neoadjuvant chemoimmunotherapy (P=0.009) had a significantly lower risk of recurrence.

**Conclusion:**

Achieving pCR does not guarantee cure. Clinical T stage and neoadjuvant regimen were independent risk factors for recurrence. Identifying high-risk patients among those with pCR was crucial for tailoring personalized surveillance and adjuvant strategies.

## Introduction

Gastric Cancer, ranking as the fifth most common malignancy and the fifth leading cause of cancer-related deaths globally, poses a significant therapeutic challenge, particularly in locally advanced stages (cT3-4/N+) (1). The paradigm shifts toward multimodal therapy, exemplified by the FLOT4-AIO trial, has established neoadjuvant chemotherapy as a standard of care achieving pathological complete response (pCR) in approximately 16% of patients (2). Despite this progress, a subset of patients who attain pCR—a strong prognostic marker associated with improved survival—still experience disease recurrence, with reported rates ranging from 5% to 15% across major clinical trials (3–6). This paradoxical phenomenon underscores the critical need to unravel the biological determinants and modifiable risk factors underlying recurrence in this ostensibly ‘low-risk’ population.

The recurrence of disease in patients who achieve pCR after neoadjuvant therapy may be influenced by various factors, such as the tumor’s molecular characteristics, the presence of microscopic residual disease post-surgery, and individual patient differences (7–9). Although several studies have explored the factors associated with postoperative recurrence in pCR patients, inconsistent conclusions have emerged due to limitations in sample size and variations in study design. Some studies suggest that lymphovascular invasion, the choice of postoperative adjuvant therapy, and changes in the immune microenvironment may be linked to recurrence risk (10, 11); however, these findings lack systematic analysis and large-scale data support. Identifying the risk factors for postoperative recurrence in pCR patients is therefore essential for optimizing postoperative management and developing personalized treatment strategies. This study retrospectively analyzed 16 patients who achieved pCR after neoadjuvant therapy but later experienced recurrence in two years after surgery, comparing them with 32 patients without recurrence. The clinical, pathological characteristics, and related factors of both groups are systematically compared. The goal is to identify potential risk factors for postoperative recurrence and provide evidence to improve postoperative management and enhance long-term survival in such kind of patients.

## Materials and methods

### Patient population

We conducted this retrospective study at Henan Provincial Cancer Hospital to identify clinical factors associated with recurrence in locally advanced gastric cancer patients who achieved pathological complete response (pCR) after neoadjuvant therapy, using data from electronic medical record systems. The trial was conducted in strict adherence to the principles outlined in the Declaration of Helsinki, and the study protocol received approval from the Medical Ethics Committee of Henan Provincial Cancer Hospital (approval number 2025-279). Since this is a retrospective study, the ethical committee approved the waiver of informed consent from the patients.

### Inclusion and exclusion criteria

The Inclusion criteria for this study as follows: 1) Histologically confirmed adenocarcinoma of the stomach or gastroesophageal junction (G/GEJ); 2) Clinically staged as T3–T4 or node-positive (N+) without distant metastasis, based on contrast-enhanced computed tomography (CT) of the chest, abdomen, and pelvis (using iopromide contrast and 1-mm slice thickness) and/or endoscopic ultrasound; 3) Indicated for neoadjuvant therapy by a multidisciplinary team specialized in G/GEJ cancer; 4) Received neoadjuvant chemotherapy, neoadjuvant chemoimmunotherapy, or neoadjuvant chemoradiotherapy; 5) Underwent curative surgery resulting in R0 resection (no residual microscopic or macroscopic tumor); 6) The postoperative pathology showed a complete pathological response (TRG 0 and no lymph node metastasis); 7) Recurrence within 2 years after surgery.

The exclusion criteria of this study as follows: 1) Presence of distant metastasis at diagnosis and underwent conversion therapy; 2) History of other primary malignancies before neoadjuvant therapy initiation; 3) Incomplete follow-up information/Loss to follow-up.

According to the inclusion and exclusion criteria described above, patients who experienced recurrence were selected and matched with non-recurrent patients at a ratio of 1:2 based on gender and age. The non-recurrent patients served as the control group, and comparative analyses were conducted between the two groups.

### Patient treatment

All patients were reviewed in MDT discussions and received treatment based on the outcomes of these discussions. The neoadjuvant chemotherapeutic regimen administered to the patients includes neoadjuvant chemotherapy, primarily the SOX (tegafur plus oxaliplatin) regimen or XELOX (capecitabine plus oxaliplatin) regimen. The neoadjuvant chemoimmunotherapy includes SOX or FLOT (calcium leucovorin, fluorouracil, oxaliplatin, and docetaxel) plus PD-1 inhibitors. For patients who received neoadjuvant chemoradiotherapy, the radiation dose administered was 45Gy/25f.

### Efficacy assessment

The clinical efficacy assessment was conducted in accordance with the RECIST 1.1 guidelines. Among the participants in this study, no patients achieved complete remission (CR) or experienced disease progression (PD). Partial response (PR) was defined as a reduction of at least 30% in the sum of the diameters of target lesions compared to baseline, with no new lesions and no progression of non-target lesions. Stable disease (SD) was defined as a situation where the reduction in target lesions did not meet the criteria for PR, nor did the increase reach the threshold for PD, placing it between the two. DFS refers to the time interval between the surgery date and the first recurrence or the last follow-up. The median follow-up time refers to the time interval between the surgery date and the last follow-up.

Histopathologic regression was evaluated using the modified Ryan Classification, with a focus on the primary tumor (12, 13). A pCR is defined as the absence of residual viable tumor cells in all resected tumor specimens, including all examined lymph nodes, as determined by hematoxylin and eosin staining.

### Statistics

SPSS version 22.0 (IBM Corp., Armonk, NY, USA) was used for all statistical analyses. Continuous variables (age, days from diagnosis to surgery, number of neoadjuvant therapy cycles, number of lymph nodes detected) were expressed as median and range, CEA, CA199, and CA724 levels at initial diagnosis and preoperation were expressed median (interquartile range, IQR). Differences between groups were assessed using the independent samples t-test for normally distributed variables, and the non-parametric Mann-Whitney test for non-normally distributed variables. Categorical variables were compared using the chi-square test or Fisher’s exact test. Cox regression analysis was performed to study the factors that affected DFS between the two groups of patients with differing clinical characteristics. Given the small number of recurrence events in 2 years (n=16) and the potential for confounding by age and sex, we performed an exploratory matched-pair analysis to identify clinical features associated with recurrence. Patients who experienced recurrence (cases) were matched in a 1:2 ratio with patients who did not experience recurrence (controls) based on sex and age ( ± 5 years). To ensure the validity of the control group during the observational period, we required that all matched controls had a minimum follow-up time of at least 3 years. After matching, differences in clinical characteristics between the case and control groups were compared using appropriate tests.

## Results

### Patients’ characteristics

**Figure 1:**
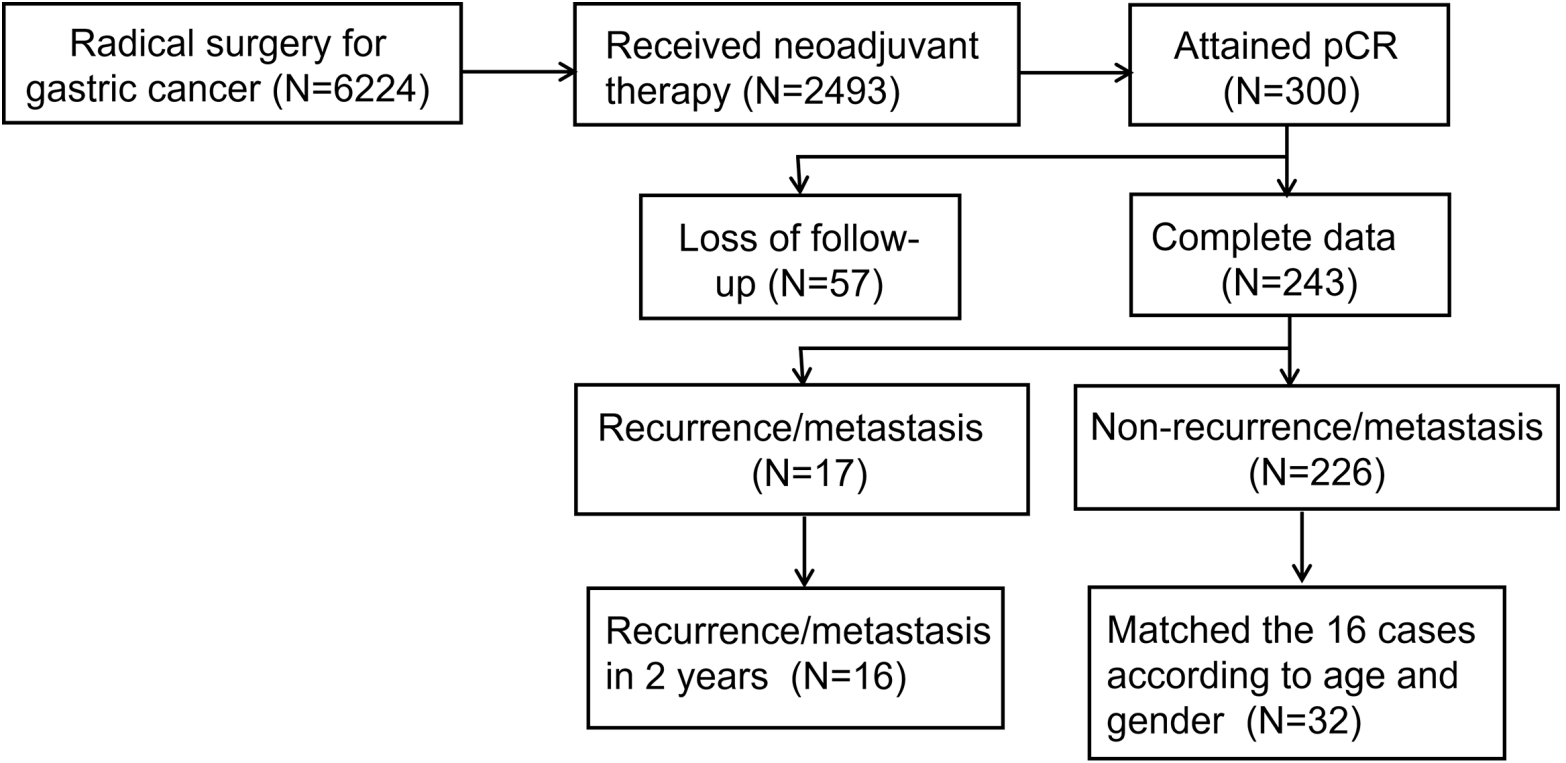
Screening procedure.

From January 2018 to December 2023, a total of 6,224 patients at Henan Provincial Cancer Hospital underwent radical surgery for gastric cancer, of which 2,493 received neoadjuvant therapy prior to surgery. Among these 2,493 patients, 300 achieved pCR, resulting in a pCR rate of 12%. Of these 300 patients, 57 were lost to follow-up. Among the remaining 243 patients, 17 experienced recurrence, yielding a recurrence rate of 7% for pCR patients. Excluding one patient with recurrence at 40.4 months postoperatively, the remaining 16 recurrent patients were included in the analysis. **Figure 1** shows the screening and inclusion process for the enrolled patients. Recurrence patients were matched with non-recurrence patients in a 1:2 ratio based on gender and age, resulting in a total of 48 patients included in this analysis. **Table 1** presents the baseline characteristics of the two groups.

**Table 1:** Patient baseline characteristics.

Characteristic		Relapsed group (N=17)	Non-relapsed group (N=34)	*P* value
Gender				1
	Male	11	22	
	Female	5	10	
Age	Median (range)	58 (38-69)	58 (10-71)	0.591
Days from diagnosis to surgery	Median (range)	89 (57-223)	88 (67-265)	0.948
Location				0.657
	GEJ	12	22	
	Gastric	4	10	
cT				0.075
	cT3	4	17	
	cT4	12	15	
cN				0.153
	cN0-1	6	19	
	cN2-3	10	13	
Grade				0.029
	G3	11	10	
	G1-2	5	22	
Her2 overexpression^**^		3	5	0.746
PD-L1 CPS^#^				0.092
	≥1	4	11	
	<1	6	3	
Neoadjuvant regimen				0.001
	Chemotherapy	4	14	
	Chemoimmunotherapy	7	18	
	Chemoradiotherapy	5	0	
No. of neoadjuvant cycles	Median (range)	3 (2-8)	4 (2-6)	0.159
Clinical efficacy of neoadjuvant therapy				0.757
	PR	5	12	
	SD	11	20	
No. of nodes detected	Median (range)	25 (2-62)	24 (11-44)	0.939
No. of perioperative cycles	Median (range)	7 (3-9)	7 (3-10)	0.585
CEA at diagnosis	Median(IQR)	3.18(1.32-8.14)	3.13(1.77-944)	0.891
CA199 at diagnosis	Median(IQR)	10.96(6.22-221.0)	12.31(6.32-87.38)	0.732
CA724 at diagnosis	Median(IQR)	9.06(2.61-17.66)	1.99(1.16-5.17)	0.009
CEA before surgery	Median(IQR)	2.08(1.68-4.05)	2.13(1.64-3.67)	0.681
CA199 before surgery	Median(IQR)	11.66(8.10-21.42)	10.50(6.11-16.32)	0.403
CA724 before surgery	Median(IQR)	5.91(1.49-16.60)	2.22(1.32-4.67)	0.045

^**^2 cases in the recurrence group and 13 cases in the non-recurrence group, Her2 expression was not tested.

^#^6 cases in the recurrence group and 18 cases in the non-recurrence group, PD-L1 CPS was not tested.

### Clinical efficacy of neoadjuvant therapy

Among the 16 patients with recurrence, 7 patients had measurable lesions according to the Response Evaluation Criteria in Solid Tumors version 1.1 (RECIST v1.1) criteria, with 5 cases achieving PR and 2 cases achieving SD. In the 9 patients without measurable lesions according to RECIST v1.1 criteria, all showed disease improvement, with a disease control rate of 100%. Among the 32 matched non-recurrent patients, 13 patients with measurable lesions according to RECIST v1.1, 12 cases achieved PR and one SD. In the 19 patients without measurable lesions according to RECIST v1.1 criteria, all showed disease improvement, with a disease control rate of 100% (detailed in **Table 1**).

### Adjuvant therapy after surgery

In the recurrence group, 2 patients did not receive postoperative adjuvant therapy; 3 patients received one cycle of adjuvant chemotherapy; 1 patient received two cycles of chemotherapy combined with PD-1 antibody therapy; 3 patients received three cycles of chemotherapy combined with PD-1 antibody therapy; and 7 patients received four cycles of adjuvant chemotherapy. In the control group, 3 patients did not receive postoperative adjuvant therapy; 1 patient received one cycle of adjuvant chemotherapy; 9 patients received two cycles of chemotherapy; 5 patients received three cycles of adjuvant chemotherapy; 5 patients received four cycles of chemotherapy; 5 patients received three cycles of chemotherapy combined with PD-1 antibody therapy; and 4 patients received three cycles of chemotherapy combined with PD-1 antibody therapy, followed by PD-1 antibody maintenance therapy for up to six months.

### Types of recurrence

The median follow-up time for the recurrence group was 41.6 (IQR 20.9-65.3) months, while the follow-up time for the control group was 53.5 (IQR 42.4-61.3) months. Among the 17 patients who initially experienced recurrence, one patient relapsed at 40.6 months post-surgery with intracranial metastasis. Based on the inclusion and exclusion criteria, this patient was excluded. Consequently, the final case cohort comprised 16 patients, all of whom experienced disease recurrence within 2 years after surgery. Notably, 11 of these 16 patients (68.8%) relapsed within the first postoperative year.

### Univariate and multivariate analysis

Univariate analysis revealed significant differences in tumor grade, neoadjuvant therapy regimen, PD-L1 expression, and preoperative CEA levels between the recurrence and non-recurrence groups (**Table 2**). Factors with a *P*-value ≤ 0.1 from the comparison in **Table 2** were selected for inclusion in the multivariate analysis using Cox regression. The results of this multivariate analysis were presented in **Table 2**. The findings suggested that the neoadjuvant therapy regimen and clinical T stage were independent prognostic factors influencing postoperative recurrence in patients who achieve pCR following neoadjuvant therapy.

**Table 2:** Univariate and Multivariate cox analysis.

Variates		Multivariate analysis		Multivariate analysis	
		HR (95% CI)	*P* values	HR (95% CI)	*P* values
Gender	Male/Female	0.915 (0.318-2.636)	0.870		
Age	≥60 / <60	1.072 (0.399-2.878)	0.891		
Location	GEJ / Gastric	1.251 (0.403-3.881)	0.698		
cT	cT3 / cT4	0.355 (0.114-1.102)	0.073	0.094 (0.010-0.852)	0.035
cN	cN0-1 / cN2-3	1.811 (0.183-1.392)	0.187		
Grade	Poorly differentiated / Moderately to well-differentiated	3.475 (1.203-10.041)	0.021	1.823 (0.305-10.89)	0.510
Her2 overexpression^**^	Yes / No	0.805 (0.225-2.888)	0.739		
PD-L1 CPS^#^	≥1 / <1	0.26 (0.072-0.934)	0.039	0.277 (0.048-1.587)	0.149
Neoadjuvant regimen*	Chemotherapy vs chemoimmunotherapy	4.77 (1.029-22.113)	0.046	0.095 (0.018-0.502)	0.009
No. of neoadjuvant cycles	≥4 vs <4	0.424 (0.158-1.140)	0.089	0.260 (0.035-1.926)	0.188
No. of perioperative cycles	≥6 vs <6	0.405 (0.151-1.088)	0.073	0.720 (0.153-3.402)	0.679
CEA levels at diagnosis	Elevated vs Normal	0.592 (0.202-1.733)	0.339		
CA199 levels at diagnosis	Elevated vs Normal	1.515 (0.518-4.436)	0.448		
CA724 levels at diagnosis	Elevated vs Normal	1.305 (0.473-3.600)	0.607		
CEA levels before surgery	Elevated vs Normal	0.111 (0.015-0.848)	0.034	0.463 (0.103-2.077)	0.315
CA199 levels before surgery	Elevated vs Normal	0.304 (0.068-1.359)	0.119		
CA724 levels before surgery	Elevated vs Normal	0.324 (0.073-1.450)	0.141		

**2 cases in the recurrence group and 13 cases in the non-recurrence group, Her2 expression was not tested.

#6 cases in the recurrence group and 18 cases in the non-recurrence group, PD-L1 CPS was not tested.

*No patients in the non-recurrence group received neoadjuvant chemoradiotherapy; therefore, patients who received neoadjuvant therapy were not included in the Cox analysis.

## Discussion

This study analyzed the clinical characteristics of gastric cancer patients who achieved pCR after neoadjuvant therapy at our center between 2018 and 2023 and later experienced disease recurrence. To eliminate the influence of gender and age, we matched patients based on gender and age during the matching process, and analyzed other clinical factors that may affect recurrence. Additionally, we compared the clinical data of non-recurrent pCR patients matched by age and gender. The findings indicated that the neoadjuvant treatment regimen and clinical T stage were independent prognostic factors for postoperative recurrence in pCR patients.

In this study, we observed a significant difference in postoperative recurrence between neoadjuvant chemotherapy combined with PD-1 inhibitors and chemotherapy alone. The combination of neoadjuvant chemotherapy and immunotherapy was associated with a significantly lower risk of recurrence (*P*=0.046) in this study. These findings highlight the potential role of immunotherapy in the neoadjuvant treatment of gastric cancer. In recent years, immunotherapy has made significant strides in treating various cancers, particularly gastrointestinal cancers like esophageal and gastric cancers (14–17). The use of immune checkpoint inhibitors, such as PD-1/PD-L1 antibodies, has demonstrated promising efficacy not only in advanced gastric cancer, but also in early gastric cancer. Studies have shown that combining immunotherapy with chemotherapy can improve both pCR and disease-free survival (DFS) in gastric cancer patients (18–22). The possible mechanisms by which the combination of chemotherapy and immunotherapy as neoadjuvant treatment reduces postoperative recurrence in cancer patients may include the following: 1) Chemotherapy can potentiate the efficacy of immunotherapy through several interrelated mechanisms. Primarily, it induces immunogenic cell death, leading to the increased release and exposure of tumor-associated antigens (23). This process enhances the ability of antigen-presenting cells, such as dendritic cells, to prime and activate tumor-specific T cells (23–25). Subsequently, immunotherapy, particularly immune checkpoint inhibitors, can effectively remove the inhibitory signals on these pre-activated T cells, unleashing a more robust and sustained cytotoxic attack against residual cancer cells (24, 25). This synergistic sequence—from chemotherapy-mediated immune priming to immunotherapy-facilitated T cell execution—may underpin the observed superior antitumor response and reduced recurrence rates in patients receiving the combined modality. 2) Chemotherapy can reduce the primary tumor burden and control micrometastasis, while immunotherapy may help maintain immune memory to recognize and eliminate residual cancer cells, thus reducing the risk of recurrence and improving long-term survival (26, 27). 3) Chemotherapy may contribute to the reduction of immunosuppressive cells, such as myeloid-derived suppressor cells and regulatory T cells, or decrease tumor burden. This could potentially transform an “immune-cold” tumor microenvironment into a more “immune-favorable” one, which is crucial for enhancing the efficacy of immunotherapy (28, 29). The findings of this study suggest that neoadjuvant chemotherapy combined with immunotherapy may reduce the risk of postoperative recurrence by enhancing immune responses and improving the tumor microenvironment. However, the effectiveness of immunotherapy may be influenced by factors such as the patient’s immune microenvironment and tumor immune evasion mechanisms (30, 31). Further research is needed to better understand the underlying mechanisms of action.

Although immunotherapy has demonstrated significant clinical benefits in some patients, many others have not experienced similar outcomes. This disparity may be attributed to factors such as individual variations in immune responses, the heterogeneity of the tumor microenvironment, and changes in immune checkpoint molecule expression (32–34). Consequently, future research should focus on identifying patient populations that are most likely to benefit from immunotherapy and further optimizing combination treatment strategies.

We also found that the clinical T stage is a key factor influencing postoperative recurrence. The tumor stage, particularly the T stage, plays a critical role in the prognosis of gastric cancer (35, 36). Clinical staging not only reflects the degree of local invasion but is also closely associated with the tumor’s biological behavior. Patients with higher T stages typically have a greater tumor burden and poorer local control, which increases the risk of postoperative recurrence (35). Even in patients who achieve pCR, the extent of local tumor invasion remains a significant factor affecting recurrence, despite the success of preoperative treatment.

Additionally, the impact of the T stage may be closely associated with the tumor’s molecular characteristics, microenvironment, and its responsiveness to treatment (36, 37). For patients with a higher clinical T stage, even if a pCR is achieved, there may still be the presence of microscopic residual lesions or immune evasion by tumor cells, which could lead to postoperative recurrence. Therefore, for patients with higher T stages, alongside conventional chemotherapy and immunotherapy, more personalized treatment strategies, such as targeted therapy or cell therapy, may be necessary to further decrease the risk of postoperative recurrence.

While this study highlights the impact of neoadjuvant therapy and clinical T stage on postoperative recurrence, several limitations remain. First, the small sample size, particularly the limited number of patients with postoperative recurrence, may affect the statistical significance of the results and the generalizability of the conclusions. Future research should aim to expand the sample size and conduct multicenter, large-scale cohort studies to validate our findings. Second, this study is based solely on clinical factors. Given the broad time span for patient enrollment, some patients did not undergo PD-L1 and/or Her2 testing, and the molecular biomarkers and immune microenvironment of tumors were not explored in depth. Future studies could integrate multi-omics data to investigate new biomarkers and therapeutic targets, facilitating the development of personalized treatment strategies. Lastly, the matched case-control analysis, while useful for generating hypotheses in this imbalanced dataset, carries the inherent limitation that control patients matched based on their non-recurrence status at the time of analysis remain at future risk.

This study underscored the potential of neoadjuvant therapy in gastric cancer and provides insights for clinical decision-making. Our findings indicated that neoadjuvant chemoimmunotherapy and clinical T stage were independent factors influencing postoperative recurrence in pCR patients. Compared to neoadjuvant chemotherapy and cT4 stage, patients receiving neoadjuvant chemoimmunotherapy and those with cT3 stage had a lower risk of recurrence. For those with high tumor burdens, particularly advanced T stages (such as cT4), more aggressive treatment and closer postoperative monitoring are recommended. Further research into recurrence mechanisms and the integration of neoadjuvant therapy, immunotherapy, and molecular targeting may improve gastric cancer prognosis.

## Data Availability

The original contributions presented in the study are included in the article/supplementary material. Further inquiries can be directed to the corresponding author.
